# Acquired Hemophilia A Post-COVID-19 Vaccination: A Case Report and Review

**DOI:** 10.7759/cureus.21909

**Published:** 2022-02-04

**Authors:** Hussam Al Hennawi, Mohammad K Al Masri, Mohamad Bakir, Mohieddin Albarazi, Feras Jazaeri, Talal N Almasri, Sami J Shoura, Abdul Rahman R Barakeh, Abdulrahman Taftafa, Muhammad K Khan, Henry I Zaleski

**Affiliations:** 1 College of Medicine, Alfaisal University College of Medicine, Riyadh, SAU; 2 Internal Medicine, John H. Stroger, Jr. Hospital of Cook County, Chicago, USA; 3 College of Medicine, Dow University of Health Sciences, Civil Hospital Karachi, Karachi, PAK; 4 Hematology and Oncology, Houston Methodist Hospital, Houston, USA

**Keywords:** hematoma, immunosuppressive therapy, acquired hemophilia a, sars-cov-2, covid-19

## Abstract

Acquired hemophilia A (AHA) is an inhibitory coagulopathy that represents a rare variant of hemorrhagic syndromes. We present a case of idiopathic AHA in a 75-year-old male patient with a cutaneous hematoma that could be attributed to a recent COVID-19 vaccination. The aim of this report is to raise awareness of a possible association between AHA and COVID-19 vaccination and to review similar reported cases and management plans to prevent the development of possible morbidity and debilitating complications. This case illustrates an exceptionally rare side effect of the COVID-19 vaccination. The advantages of obtaining the COVID-19 vaccine outweigh the risks.

## Introduction

Hemophilia is the most prevalent and severe hemorrhagic disorder [1]. Hemophilia can be hereditary or acquired, with the latter occurring less frequently. Autoantibodies that form against a coagulation factor cause acquired hemophilia. Such antibodies develop primarily against factor VIII, referred to as “acquired hemophilia A” (AHA) [2]. Acquired hemophilia is rare, with an annual incidence of 1.5 per million people [3]. However, the frequency changes with age, ranging from 0.045 per million per year in children under the age of 16 to 14.7 per million per year in individuals over the age of 85. As a result, adults are more likely to develop the disease than children [4, 5]. Because of its rarity and the complexities of the laboratory workup, it is frequently challenging to diagnose a patient with AHA. Clinically, the condition ranges from life-threatening hemorrhage at one end of the spectrum to mild or no bleeding at the other, and the onset of life-threatening bleeding is most common during the first few weeks after developing the condition. However, it can occur at any time if not managed appropriately [3-5]. AHA has been linked to autoimmune disorders, medications, tumors, lymphoproliferative cancers, and infections [6]. A thorough history of immunological disorders, medications, and the wide spectrum of related medical problems that predispose the patient to acquired hemophilia should be obtained [3]. Patients with recent onset of abnormal bleeding, particularly the elderly and peripartum women, should be evaluated for AHA [4]. On physical examination, affected patients may present with widespread spontaneous subcutaneous hematomas, without a precipitating factor [4]. The rarity of this condition may account for the considerable delay in the diagnosis, so a thorough physical examination along with the necessary laboratory tests should be performed when the history reveals an etiological cause [1]. In this paper, we present a case of AHA following the COVID-19 vaccination.

## Case presentation

**Figure 1:**
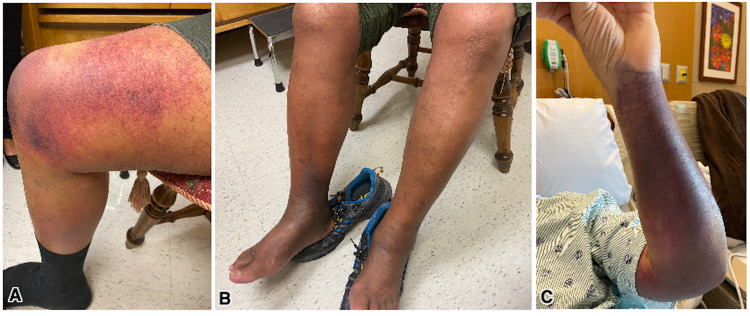
Spontaneous ecchymosis involving right thigh (A), and right ankle (B) rendering the leg double the normal circumference. Left upper extremity ecchymosis had spontaneously developed despite immunosuppressive treatment (C).

A 75-year-old gentleman with a past medical history of hypertension, dyslipidemia, coronary artery disease, and benign prostatic hyperplasia was referred due to a possible coagulopathy following COVID-19 vaccination, accompanied by bleeding into the soft tissues, distinct ecchymoses, possible compartment syndrome, and anemia secondary to soft tissue bleeding. Bleeding into the skin began two months prior to presentation and approximately three months following the second dose of (BNT162b2, Comirnaty, Pfizer/BioNTech) COVID-19 vaccine, which at first involved the right forearm followed by the right flank region. The patient reported complete resolution of the complaint; however, this was followed by severe bleeding into the right lower extremity rendering the leg double the normal circumference (Figures 1A, 1B). At that time the patient was unable to raise his leg concerning possible compartment syndrome.

The review of systems showed no constitutional complaints. The patient denied having cough, chest pain, shortness of breath, and mucosal bleeding diathesis. No other significant ecchymoses were present except for the residual presence of ecchymoses in the right flank. The physical exam was otherwise unremarkable. Initial workup showed activated partial thromboplastin time (aPTT) of over 90 seconds (normal range = 23-36 seconds). He was admitted to the hospital for further evaluation and treatment. Other laboratory investigations were significant for: red blood cells 2.45 m/µL (normal range = 4.4-6.0 m/µL), hemoglobin 7.4 g/dL (normal range = 14-18 g/dL), lupus anticoagulant-sensitive PTT 114.3 seconds (normal range = 27-38 seconds), D-dimer 8.22 µg/mL FEU (normal range = 0.00-0.40 µg/mL FEU). Venous duplex ultrasound of the lower extremities was subsequently performed, which demonstrated no evidence of deep venous thrombosis (DVT). Ankle-brachial index (ABI) and toe/brachial index (TBI) testing was done to screen for lower extremity arterial thrombosis, where both indices fell into the normal range bilaterally. The patient underwent further laboratory studies consisting of factor VIII activity that showed a severely low level of less than 1% (normal range = 60%-150%), an extremely elevated Bethesda assay of 318 Bethesda units (BU), normal von Willebrand factor antigen level, and a 1:1 mixing study which failed to correct elevated aPTT. Based on preliminary investigations, a diagnosis of factor Vlll inhibitor was made and an appropriate treatment plan was suggested.

The treatment plan was initiated with recombinant factor Vlla (rFVIIa; NovoSeven®, Novo Nordisk, Denmark) given at 8,000 units every three hours. This was continued for multiple days until some initial improvement in the aPTT was found. However, factor Vlll activity remained low for multiple days despite factor Vlla administration. Due to unsatisfactory improvement of factor Vlll activity, desmopressin (DDAVP) was given for a short period of less than a week but was later discontinued due to significant hyponatremia below 125 mEq/L (normal range = 135-148 mEq/L). Intermittent courses of prednisone 80 mg daily for three days were started. There was some mild improvement in the aPTT to 50 seconds measured following factor Vlla injections. The patient continued to show minimal improvement, and a course of rituximab 375 mg/m^2^ weekly for four consecutive weeks was initiated. Nevertheless, no discernable aPTT improvement was appreciated. Despite the treatment course, the patient was found to have diffuse left forearm ecchymoses (Figure 1 C). He also developed sudden lower extremity pain accompanied by an elevation in D-dimer level, which necessitated an urgent investigation for DVT and pulmonary embolism (PE). Duplex ultrasound of the lower extremities was negative for DVT, and computed tomography angiogram of the chest was negative for PE. A week later, a course of intravenous cyclophosphamide 750 mg/m^2^ was initiated. Following the treatment course, factor Vlll activity was found at 20%, with a subsequent decrease to 10% was noted. Treatment with cyclosporine 25 mg twice daily was added, and restoration of Vlll activity to 20% was achieved. At this point in time, the patient was stable and had no new evidence of bleeding ecchymoses at any site, with an aPTT of 33.6 seconds. He was discharged in a stable condition and continued on cyclosporine 25 mg twice daily for one month and to be seen weekly as an outpatient.

## Discussion

AHA is a rare bleeding disorder characterized by autoantibodies against clotting factor VIII that is associated with increased morbidity and mortality and primarily affects older adults with no prior personal or family history of bleeding [7]. Approximately half of the patients with AHA have no underlying or predisposing conditions (idiopathic cases), and the remaining instances are related to a variety of conditions (for example, autoimmune and dermatologic disorders, postpartum, infections, cancers, and medications) [8]. AHA is characterized by bleeding into the skin, muscles, soft tissues, and mucous membranes in more than 80% of cases [8]. The pathophysiology of AHA is unknown; however, T lymphocytes and certain genetic polymorphisms are thought to have a role [9]. Vaccines have long been linked to the production of autoantibodies. It has been proposed that vaccination may cause an autoimmune response due to antigenic mimicry as well as stimulation of dormant autoreactive T and B cells [10]. When history and physical examination raise suspicions, a complete blood count and coagulation profile should be performed to confirm the diagnosis of acquired hemophilia [1]. The diagnosis is frequently delayed due to a lack of recognition of this rare condition by clinicians who are unfamiliar with the disorder, therefore, the diagnosis must be made in the context of a single prolonged aPTT with an insufficient correction following a 1:1 mixing analysis [11]. Once a low FVIII level is established, and there is confirmation of the presence of FVIII inhibitor (INH) the diagnosis is confirmed [11].

Treatment of AHA has two major goals: hemostasis and elimination of the inhibitor. The objective is to manage life-threatening bleeding while limiting invasive procedures to reduce the danger of additional bleeding [3,12]. Not all bleeding requires hemostatic therapy. If the bleeding is mild and does not involve a crucial organ or region, observation may be considered [11]. In the case of severe acute bleeding, bypass treatment is the first-line management. It can be used as a preventative measure before invasive procedures to prevent bleeding in patients who are presumed to be particularly vulnerable as well [3,11,12]. Currently, two bypassing agents are available to maintain hemostasis: activated prothrombin complex concentrates (APCC) and recombinant activated factor VII (rFVIIa), which are still the most effective agents available today, according to published expert guidelines [3,13,14]. Human FVIII concentrate can be used to treat hemostasis in patients with AHA, although its use is limited. It is an option for patients with low inhibitor titers. It can also be used if bypassing agents are not readily available [3,11,13]. In the existence of the autoantibodies, the patient is always at risk of life-threatening bleeding, and eradicating the inhibitor completely is critical for enhancing the patient’s survival. Furthermore, inhibitor eradication therapy should be commenced concurrently with hemostatic therapy, as delays have been linked to poor patient outcomes [1]. Prednisone (1 mg/kg/day) is the first-line treatment for this purpose. It can be taken alone or in combination with cyclophosphamide (50 to 100 mg/day), while data suggest that combination therapy is associated with improved patient results [1]. 

Our case represented a challenging course of treatment, as our patient did not show signs of improvement despite the initiation of immunosuppressive therapy. While other identified cases demonstrated improvement in factor VIII activity and resolution of skin hematomas (Table 1), our patient received advanced immunosuppressive therapy (cyclophosphamide, cyclosporine) to achieve optimal control. This could be related to the inhibitor load and the Bethesda assay, as our patient had an initial 318 Bµ/mL, which could have required an increase in treatment compared to similar cases with a milder elevation. The goal of this case report is to help raise awareness among health care professionals about a potential unusual adverse reaction associated with the COVID-19 vaccine and to illustrate the need for increased monitoring and surveillance globally to understand the association between acquired hemophilia and COVID-19 vaccine to rule out any fatal complications that may emerge.

**Table 1:** Overview of cases of acquired hemophilia A attributed to the COVID-19 vaccines. APCC = activated prothrombin complex concentrate; FEIBA = Factor VIII inhibitor bypass activity; CR = current.

Author	Ref.	Age and sex	Vaccine	First Dose	Second Dose	PTT (sec)	PTT 1:1 mix 0	Bethesda titer	Factor VIII Activity	Treatment
Al Hennawi et al.	CR	75, M	Pfizer-BioNTech SARS CoV-2 mRNA vaccine	Uneventful	Multiple sites cutaneous hematoma	89.2	Typical for delayed acting inhibitor of coagulation	318 Bµ/ml	<1%	rFVIIa followed by prednisone 80 mg daily for 3 days, rituximab 375 mg/m^2^, cyclophosphamide 750 mg/m^2^, cyclosporine 25 mg.
Radwi and Farsi	[15]	69, M	Pfizer-BioNTech SARS CoV-2 mRNA vaccine	Cutaneous hematoma	Multiple sites cutaneous hematoma and intramuscular hematoma	115.2	Immediate near correction at 45 seconds	80 Bµ/mL	1%	Prednisone (1 mg/kg) for 4 weeks followed by 5% rFVIIa.
Cittone et al.	[16]	85, M	Moderna COVID-19 (mRNA-1273) vaccine	Multiple sites hematoma and bilateral knee bleeding	Abdominal hematoma	49	Typical for delayed acting inhibitor of coagulation	2.2 Bµ/mL	Not detectable	rFVIIa then switched to APCC + prednisone 100 mg/d + rituximab.
Cittone et al.	[16]	86, F	Moderna COVID-19 (mRNA-1273) vaccine	Uneventful	Not reported	Prolonged	Typical for delayed acting inhibitor of coagulation	1.01 Bµ/mL	23%	rFVIIa + APCC for control of local bleeding + prednisone (1 mg/kg) FVIII:C increased to 178% after 17 days.
Cittone et al.	[16]	72, F	Moderna COVID-19 (mRNA-1273) vaccine	Extensive cutaneous hematoma	Not reported	184	Typical for delayed acting inhibitor of coagulation	12.4 Bµ/mL	Not detectable	rFVIIa+ tranexamic acid + prednisone (100 mg/d) + rituximab 375 mg/m^2^ weekly (4 doses). Bleeding tendency improved 1 week after the first and third dose of rituximab. FVIII activity increased to 5%, while FVIII inhibitor decreased to 5.6 BU/ml.
Portuguese et al.	[17]	76, F	Moderna COVID-19 (mRNA-1273) vaccine	Uneventful	Multiple sites cutaneous hematoma and melanotic stool	122	N/A	11.2 Bµ/mL	<3%	vWF/FVIII replacement therapy with Humate-P 2290 U/12 hours × 4 doses IV immunoglobulin (IVIg) + methylprednisolone 125 mg. Marked elevation of vWF and factor VIII with inhibitor levels <0.5 BU after 2 weeks of treatment.
Farley et al.	[18]	67, M	Pfizer-BioNTech SARS CoV-2 mRNA vaccine	Uneventful	Multiple sites cutaneous hematoma	72	No significant correction	110 Bµ/mL	Not detectable	FEIBA at 4,500 µ/kg/8 hours, oral prednisone 90 mg, and rituximab (375 mg/m^2^/week x 4 doses). After the second dose of rituximab, FEIBA stopped at 8 Bethesda units/ml.

## Conclusions

AHA is a relatively rare variant of hemorrhagic syndrome with a challenging diagnosis. Our case report and literature review may add to the evolving reports of immune-mediated conditions that develop after COVID-19 vaccination, particularly the association between the COVID-19 vaccine and AHA. To date, no clear association has been established between these vaccines and autoimmune disorders, which can be challenging due to the variable timeline of presentation and manifestation of possible secondary autoimmune processes. This case exemplifies a very unusual side effect of vaccination. Our case does not affirm causality, and further studies to investigate this possible rare event may be necessary. However, given the current situation, the advantages far outweigh the risks; therefore, there should be no hesitation to seek vaccination. We aim to increase awareness of possible associations and encourage surveillance of bleeding manifestations, especially during the window of early weeks after the administration of both doses.
